# Comprehensive exploration of graphically defined reaction spaces

**DOI:** 10.1038/s41597-023-02043-z

**Published:** 2023-03-20

**Authors:** Qiyuan Zhao, Sai Mahit Vaddadi, Michael Woulfe, Lawal A. Ogunfowora, Sanjay S. Garimella, Olexandr Isayev, Brett M. Savoie

**Affiliations:** 1 Davidson School of Chemical Engineering, Purdue University, West Lafayette, IN 47906 USA; 2 Department of Chemistry, Purdue University, West Lafayette, IN 47906 USA; 3 Department of Chemistry, Carnegie Mellon University, Pittsburgh, PA 15213 USA

**Keywords:** Reaction kinetics and dynamics, Reaction mechanisms

## Abstract

Existing reaction transition state (TS) databases are comparatively small and lack chemical diversity. Here, this data gap has been addressed using the concept of a graphically-defined model reaction to comprehensively characterize a reaction space associated with C, H, O, and N containing molecules with up to 10 heavy (non-hydrogen) atoms. The resulting dataset is composed of 176,992 organic reactions possessing at least one validated TS, activation energy, heat of reaction, reactant and product geometries, frequencies, and atom-mapping. For 33,032 reactions, more than one TS was discovered by conformational sampling, allowing conformational errors in TS prediction to be assessed. Data is supplied at the GFN2-xTB and B3LYP-D3/TZVP levels of theory. A subset of reactions were recalculated at the CCSD(T)-F12/cc-pVDZ-F12 and *ω*B97X-D2/def2-TZVP levels to establish relative errors. The resulting collection of reactions and properties are called the Reaction Graph Depth 1 (RGD1) dataset. RGD1 represents the largest and most chemically diverse TS dataset published to date and should find immediate use in developing novel machine learning models for predicting reaction properties.

## Background & Summary

Data scarcity is a major bottleneck for training machine learning (ML) models relevant to predicting reaction properties like transition state (TS) geometries, activation energies, and reaction mechanisms. Even when sufficiently numerous data are available, a lack of broad reaction representation still limits transferability and generalizability^1^. As deep learning methods displace traditional ML models like linear regression and support vector machines (SVMs) in chemical property prediction, the need increases for comparably large, diverse, and accurate reaction databases^2^. Despite the prevalance of quantum chemistry derived molecular property databases, comparable efforts for developing large reaction and TS databases have been frustrated by three factors. First, locating transition states solely based on reactant and product information is up to two orders of magnitude more expensive than searching for the minimum energy geometries of individual molecules^3^. This limits naive brute force approaches to enumerating reactions and searching for transition states. Second, even when a TS is successfully located and verified to contain only one imaginary frequency, verifying the reaction that the TS corresponds to and whether the TS represents a kinetically-relevant conformation require further tests that are costly and typically omitted. Recent automated reaction prediction studies report intended rates varying from 4% to 60%^4–6^ meaning that depending on the algorithm up to 96% and 40%, respectively, of the TSs that are discovered do not correspond to the reaction that was used to seed their search. Likewise, a recent study that characterized a set of 124 unimolecular decomposition reactions using conformational sampling found an average 5.2 kcal/mol deviation across different TS conformations^6^. Third, the theoretical size of reaction space is larger than molecular space, making reaction sampling a critical aspect of dataset curation. Together, these factors have contributed to the relative scarcity of TS datasets and the comparative lack of development of ML models for predicting reaction properties.

Despite these challenges, some reaction datasets with transition state geometries and activation energies have started to appear. Grambow *et al*. recently reported *ω*B97x-D3 level transition states for 12,000 reactions involving C, H, O, and N atoms^2^. This database was built using automated reaction enumeration and characterization, with a focus on expanding the diversity of reaction classes represented. The BH9 dataset, developed by Prasad *et al*. to benchmark the accuracy of different levels of theory for transition states, consists of 449 organic reactions computed at the DLPNO-CCSD(T) level of theory^7^. Rudorff *et al*. published a dataset of 4000 distinct TSs at the DF-LCCSD level for E2 and S_N_2 reactions involving a small number of nucleophiles and electrophiles^8^. These datasets have spurred the development of ML approaches to predict activation energies^9–12^ and transition state geometries^13–15^. Despite these welcome advances, these datasets remain small in comparison with the 100 k-1 m distinct records found in many molecular property databases. BH9 consists of high-accuracy post-HF calculations but includes only a few hundred TSs. The Rudorff dataset has thousands of distinct TSs but for only a narrow class of nucleophiles and electrophiles. The Grambow dataset is broader in reaction scope but limited to reactants composed of no more than seven heavy atoms and contains no conformational diversity.

To address the diversity and scarcity challenges of extant reaction datasets, we report a database of 176,992 reactions covering C, H, O, and N containing molecules up to 10 heavy atoms. This dataset has been curated using the concept of graphically defined model reactions with a uniqueness out to one bond away from the reacting atoms (i.e., a graphical depth of one). For this reason, it is named the Reaction Graph Depth 1 (RGD1) dataset. Compared with previously published datasets, RGD1 contains more reactions (15-fold and 45-fold larger than the Grambow and Prasad datasets, respectively), more complex reactions (57.8% reactions have more than seven heavy atoms) and covers more diverse reactions due to the generic enumeration rules and PubChem origin of all reactions and reactants. These reactions were characterized by our recently developed reaction prediction package, Yet Another Reaction Program (YARP)^5^, with semi-empirical GFN2-xTB^16^ and B3LYP-D3/TZVP functional and basis set as low and high level of theory, respectively, to explore the potential energy surface. The raw output files of growing string method (GSM)^17–19^, geometry optimizations, frequency calculations, and intrinsic coordinate calculations (IRC) are provided along with atom-mapped SMILES, geometries of reactant, product and transition states, activation energies, and enthalpies of reaction. The increased size and accuracy of the RGD1 TSs should facilitate the development of more general and accurate ML models for TS characterization and prediction, and further open up a new avenue for automated reaction network prediction. Although currently RGD1 only includes uncatalyzed reactions, the ongoing development of transition state automation will enable comparable catalyzed reaction databases to be built in the near future^20–22^.

## Methods

**Fig. 1:**
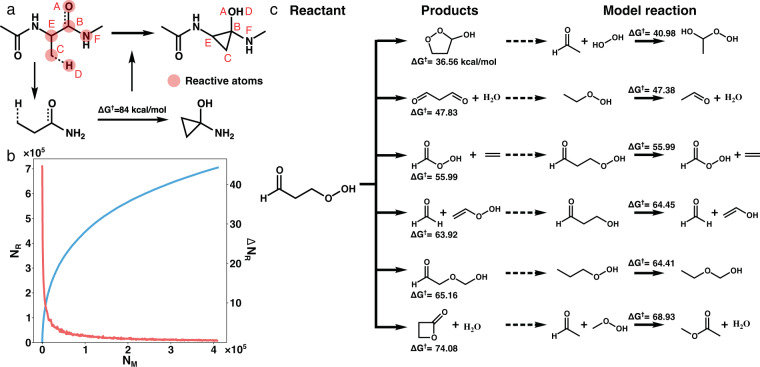
Overview of the reaction data generation process. (**a**) An illustration of generating a graphically defined model reaction from a larger reacting molecule. (**b**) The number of cumulative (blue) and new (red) model reactions generated as the 413,519 initial PubChem reactants are processed. (**c**) A subset of the model reactions generated by enumerating elementary reactions of *γ*-ketohydroperoxide (KHP).

The reaction dataset was generated in five steps. Details on each step are provided in following sections, but summarized here for convenience. First, 413,519 C,H,O,N-containing neutral closed-shell molecules with no more than ten heavy atoms were curated from PubChem^23^. Second, a graphically-defined elementary reaction step (ERS) was applied to those 413,519 molecules to enumerate potential reactions. For each enumerated reaction, a graphically defined model reaction was formed consisting of a truncated version of the reactants and products that preserved the hybridization of the reaction centers (Fig. 1a). Enumeration and conversion to model reactions resulted in ~708 k unique model reactions (Fig. 1b). Third, conformational sampling was performed on all reactant-product pairs to generate three-dimensional geometries as an input for double-ended TS searches. This step yielded a varying number of conformations for each reactant-product pair depending on the degrees of freedom and ranking by a classifier^24^, resulting in 1,339,887 total reaction conformations. Fourth, Yet Another Reaction Program (YARP) was used to localize transition states for all 1,339,887 reaction conformers at the B3LYP-D3/TZVP level of theory. Fifth, the localized TSs were filtered based on whether they correspond to reactions connecting the intended reactants and products. After a series of filtering steps to remove duplicated conformations, the final dataset contains one or more validated transition states, among other properties, for 126,857 distinct reactions (33,032 of these have two or more TSs leading to a total of 176,992 reactions with TSs). The details of each step are provided in the following sections.

### PubChem data curation

PubChem data was extracted in September 2021 through FTP service. All chemical structure data were processed using the OpenEye chemistry toolkit^25^. Standardizer was used for structure canonicalization. The cleaning of salts, removal of mixtures, inorganics, and organometallics was performed using Instant JChem software (version 20.2, ChemAxon)^26^. InChI keys were used to de-duplicate replicated structures. The dataset was further curated according to a previously described protocols^27^. All C,H,O,N-containing neutral closed-shell molecules with ten or fewer atoms were taken from this curated PubChem dataset, yielding 413,519 reactants for reaction enumeration.

### Elementary reaction step enumeration

The graphically-defined elementary reaction step (ERS) was developed in YARP to comprehensively explore the reaction space without the use of specific chemical heuristics^5^. For neutral closed-shell organic systems, the simplest ERS that yields non-trivial products is “breaking two bonds and forming two bonds” (b2f2) (Fig. 1a). This ERS has been extensively benchmarked showing that it achieves a balance of computational cost and reaction coverage in exploring reaction networks^5,6^. For a given reactant, the bond-electron (BE) matrix is computed and every combination of two different bonds are iterated. Without loss of generality, taking Fig. 1a as an example, the bonds between (A,B) and (C,D) are selected to break and two possible new bond rearrangements, {(A,C), (B,D)} and {(A,D) and (B,C)}, are considered (the latter corresponds to the reaction shown in the Fig. 1a). Thus, many b2f2 reactions may be possible for a given reactant. Here, the reaction space consisting of all b2f2 reactions possible for the 413,519 C,H,O, and N-containing molecules curated from PubChem were comprehensively explored.

### Model reaction generation

Even with strict adherence to the b2f2 formalism, the possible reaction space is enormous because tens to thousands of reactions can be enumerated for each reactant and a large fraction of them are similar. To reduce the reaction number while prioritizing distinct reactions, the concept of a graphically-defined model reaction was used. The model reaction is defined as the smallest reaction involving the same number and type of bond changes, where the types of bonds are determined by the bonded neighbors out to a specific depth. Here a depth of one was used, meaning that the bond types are unique out to their immediate bonded neighbors. This is analogous to prior definitions of models compounds for generating force-fields^28^ and parameterizing increment theories^29^. The model reaction is formed by truncating the parent reaction at the specified graphical depth, then hydrogenating the structure to a level that preserves the hybridization of the reacting atoms in the reactant and product.

A specific example is shown in Fig. 1a for a diamide cyclization. First, the reactive atoms (atom A-D in Fig. 1a) and their nearest neighbors (atom E and F) are retained to form a preliminary model reactant. The under-coordinated neighboring atoms (atom E and F) resulting from truncation are hydrogenated to preserve the local Lewis structure of the original reactant. Third, the model product is generated from the model reactant by applying the ERS (i.e., [A,B], [C,D] to [A,D], [B,C]). In this example, the model reaction results in a reduction of four heavy atoms that generalizes to mono-amides.

The total number of model reactions was tracked at intervals of every ten reactants processed at random from PubChem (Fig. 1b). The first ten reactants produced 1224 model reactions, while the last ten produced no new model reactions, which indicates a saturation of the reaction space. Considering a more specific example, 6 out of 34 b2f2 reactions of *γ*-ketohydroperoxide (KHP) are compared with their model reactions (Fig. 1c). Although these six reactions were generated from the same reactant (KHP), they yield model reactions with distinct reactants, covering both unimolecular and bimolecular reactions. As this case shows, for an individual reactant the use of model reactions does not substantially reduce the total number of possible b2f2 reactions, only their complexity. However, across the space of 413,519 reactants, the use of model reactions leads to a substantial overall reduction due to the recurrence of reaction subgraphs. The total number of unique model reactions resulting from comprehensive b2f2 enumeration of the PubChem reactants was 707,762.

### Transition state characterizations

The TS search for the enumerated reactions is the most time and resource intensive step of the reaction database generation. Here, the TS searches were performed with the recently developed reaction prediction package, YARP (v2.0), which dramatically reduces the number of DFT-level gradient calls and increases the success rate of TS searches^6^. Based on our recent benchmark studies, barrier heights are strongly dependent on the quality of the TS algorithm and conformational sampling protocols^24^. To obtain the most stable TSs for each reaction and introduce conformational diversity into the database, our recently developed conformational sampling algorithm was applied to generate up to three reactant-product conformations for each model reaction (the exact number depends on the size of the reacting system, the number of degrees of freedom, and ranking of the resulting conformations)^24^. In brief, reactant and product conformations were sampled by the Conformer-Rotamer Ensemble Sampling Tool (CREST)^30^, then the corresponding product and reactant geometries were fed to a joint-optimization algorithm to form well-aligned reactant-product pairs (i.e., reaction conformations). The sampled reaction conformations are then screened by a geometry-based random forest (RF) model to select up to three conformations that are most likely to locate an “intended” TS (i.e. the TS corresponds to the inputted reaction). In this process, unphysical enumerated reactions, such as long-distance fragment roaming and steric clashes, were excluded because no reaction conformation could pass the RF classifier. In total 589,394 reactions and 1,339,887 conformations were generated by this step as a starting point for TS localization.

The TS search in YARP consisted of four steps. First, the growing string method (GSM) was used to construct a minimal energy pathway (MEP) at the GFN2-xTB level of theory. The highest energy node along the MEP was used as an initial guess for Berny TS optimization^31^ at the same level of theory to locate an exact TS on the GFN2-xTB potential energy surface (PES). After this step, TSs with none or more than one imaginary frequency were removed from further consideration. Third, intrinsic reaction coordinate (IRC) calculations were applied to identify the reactant(s) and product(s) corresponding to the remaining TSs. The results of the IRC analysis were then compared to the enumerated reactions, and only TSs with IRC endpoints matching at least one of the reactant(s) or product(s) of the enumerated reactions were retained. As discussed below, retaining reactions involving these unexpected reactants or products, results in substantial reaction diversity beyond b2f2 in the final dataset. A total of 407,125 TSs passed this filter. Fourth, these TSs were used as initial guesses for Berny TS optimizations performed at the B3LYP/TZVP level of theory^32,33^ with D3 dispersion^34^ to obtain DFT-level TS geometries and energies.

### Reaction verification

The most straightforward way to reveal the reactant(s) and product(s) associated with an optimized TS is an IRC calculation. However, performing DFT-level IRC calculations is very time-consuming. Here, a ML model provided by YARP was utilized to replace IRC calculations and predict the reactant(s) and product(s) for 407,125 TSs in an efficient manner. The ML model uses a geometry-based featurization of the target reaction and the imaginary frequency mode of the TS and predicts whether the TS corresponds to the target reaction (intended TS) or not (unintended TS) based on a XGBoost classifier^35^. The precision of this ML model reaches 0.96 in a small testing set, which is better than previously reported models that classify the TS solely based on the largest bond length change in the imaginary frequency mode^6^. Due to the low- and high-level TS search process, there are two possible reactions that can be used as the input for the classifier, one is the original enumerated reaction and the other one is the xTB-level IRC result (in some cases these two reactions are the same). Intended scores (i.e. the probability of intended provided by the XGBoost model) were computed for each reaction and the one with higher score was reported as the reaction corresponding to the TS. If both intended scores were lower than 0.5, the confidence of the model was judged to be too low and the reaction was discarded. In total 227,115 reactions (including different conformations) were classified as intended out of 407,125 DFT-level optimized TSs, of which 118,712 were the original enumerated b2f2 reactions and 108,403 were reactions corresponding to more diverse reaction types identified by the GFN2-xTB IRC calculations.

### Calculating reaction properties

The thermochemical properties of the reactants and products are required to obtain the activation energies and enthalpies of reaction. To reduce the conformational complexity of multi-molecular reactants/products, the molecules comprising the reactants and products were individually optimized. Auto3D was applied to generate 3D geometries for the 136,881 distinct molecules parsed from 227,115 reactions^36^. Although the reactants and products of enumerated reactions are all neutral closed-shell species, this argument no longer holds when considering the reactions identified by xTB-level IRC calculations. To ensure the consistency of the database, 5,506 reactions involving radical or ionic species were discarded. In addition, some xTB-level IRC endpoints that were unstable at the DFT level, which caused changes in connectivity after Auto3D geometry generation, were also excluded. A total of ~12,000 molecules were removed by these filters. Performing B3LYP-D3/TZVP level geometry optimization on the 125,234 remaining molecules, 123,088 molecules were successfully optimized with no change in connectivity. Activation energies of forward and backward reactions were calculated by taking the difference of the TS single point energies and the reactant and product single point energies, respectively. Similarly, enthalpies of reaction were computed by taking the difference of the enthalpies of product and reactant. The thermodynamic corrections to these energies were calculated using harmonic vibrational analysis as implemented in Gaussian 16^37^. Finally, reactions with anomalous activation energies (less than 0 or greater than 500 kcal/mol) due to abnormal geometry optimization, and reactions with identical activation energies due to duplicated TS conformations were excluded.

## Data Records

All RGD1 data is free and publicly accessible on figshare^38^. Gaussian output files of TS optimizations and frequency calculations are available for 126,857 distinct reactions (33,032 of these have two or more TSs leading to a total of 176,992 reactions with TSs) at B3LYP-D3/TZVP level of theory. The raw output files are stored in a compressed archive file, RGD1.tar.gz, that is available upon request. The output files are named as MR_X_Y-TS.out, where X refers to the model reaction index (ranging from 1 to 707,762) and Y refers to the conformation index (ranging from 0 to 2).

In addition to the raw data files, a csv file and a HDF5 file^39^ are also provided for more convenient usage. The csv file named RGD1CHNO_smiles.csv contains enthalpies of reaction, atom-mapped SMILES, activation energies for each reaction. The columns of the csv file are explained in Table 1. The HDF5 file contains the geometry information and can be iterated by a python script (parse_data.py), which is provided in a publicly accessible GitHub repository^40^. The property names are provided in Table 2, along with the corresponding dictionary keys and units. The Usage Notes section provides more details about how to access the data from the csv and HDF5 files.

**Table 1:** A description of the columns in the csv file.

Column	Description
Rind	Reaction index
Rsmiles	Atom-mapped smiles of reactant(s)
Psmiles	Atom-mapped smiles of product(s)
DE_F	Activation energy of the forward reaction
DE_B	Activation energy of the backward reaction
DG_F	Free energy of activation of the forward reaction
DG_B	Free energy of activation of the backward reaction
DH	Enthalpy of reaction (forward reaction)

Energies are in kcal/mol.

**Table 2:** Data layout in the provided HDF5 file.

Property	Key	Units
Reactant smiles	Rsmiles	
Product smiles	Psmiles	
Reactant single point energy	R_E	Hartree
Reactant enthalpy	R_H	Hartree
Reactant Gibbs free energy	R_F	Hartree
Product single point energy	P_E	Hartree
Product enthalpy	P_H	Hartree
Product Gibbs free energy	P_F	Hartree
Transition state single point energy	TS_E	Hartree
Transition state enthalpy	TS_H	Hartree
Transition state Gibbs free energy	TS_F	Hartree
Reactant geometry	RG	Å
Product geometry	PG	Å
Transition state geometry	TSG	Å

## Technical Validation

### Analysis of reaction features

**Fig. 2:**
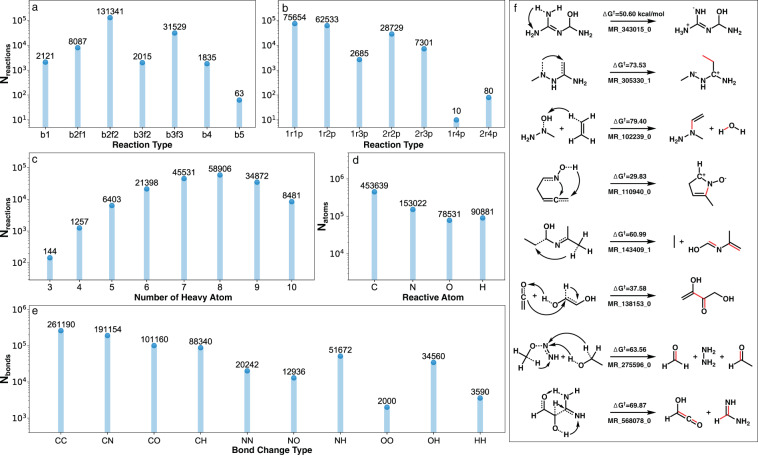
Distribution of different reaction features of the RGD1 reaction dataset. (**a**) Distribution of reactions classified by bond changes (b1, b4 and b5 reactions include both bnfn and bnf[n-1] types of reactions). (**b**) Distribution of reactions classified by the number of reactants and products, where *m*r*n*p refers to a reaction involving *m* reactants and *n* products. To avoid double counting the forward and reverse versions of each reaction, only the direction that resulted in *n* ≥ *m* was histogrammed. (**c**) Distribution of reactions classified by the number of heavy atoms. (**d**) Occurrence of each element as a reactive atom in the RGD1 reactions. (**e**) Number of times each bond type was formed or broken in the RGD1 reactions. Single, double and triple bonds are grouped together in this histogram. (**f**) Selected reactions representing b1f1, b2f1, b2f2, b3f2, b3f3, b4f4, b5f5 and b6f6 reactions (from top to bottom). The bonds represented by dotted lines on the reactant side refer to the bonds that break in the reaction and the formed bonds are represented by red lines on the product side. Note that some reactive bonds involving hydrogen are implicit and not directly drawn in these  chemical structures.

A high-level view of the diversity of RGD1 is illustrated by five reaction features, including the elementary reaction type, number of reactants and products, number of heavy atoms, reactive atoms, and types of bonds involved in the reactions (Fig. 2). Although the original reaction enumeration only contained b2f2 reactions, including unintended reactions revealed by xTB-level IRC calculations dramatically increased the diversity of ERSs represented in the database. Reactions corresponding to 11 distinct ERSs occur in the reaction database, including 6 bnfn reactions (n from 1 to 6) and 5 bnf(n-1) reactions (n from 1 to 5). The b6f6 reaction is excluded from the Fig. 2a histogram because it appears only once. The bnfn and bnf(n-1) reactions for *n* = 1,4,5 are combined into one type of reaction (bn) due to their smaller number of occurrences. The most common reaction type is b2f2, accounting for 74% of the reactions, followed by b3f3 reactions, accounting for 18% of the reactions (Fig. 2a). The other nine types of reactions account for 8% of the reactions, with the more complex reactions (i.e., b4, b5, and b6 reactions) contributing 1%. For reference, it is usually preferable to decompose multiple bond rearrangements into sequential steps involving multiple TSs. The discovery of TSs corresponding to such complex rearrangements (without intervening TSs) is thus surprising and reflects the diversity of the reaction exploration algorithm used to generate RGD1. Examples of eight ERS types included in RGD1 are selected for b1f1, b2f1, b2f2, b3f2, b3f3, b4f4, b5f5 and b6f6 reactions (Fig. 2f). The reactions in RGD1 can also be classified based on the number of reactant(s) and product(s) in each reaction. The three most common reactions in RGD1 involve one reactant and one product (1r1p, unimolecular isomerization), 1r2p (i.e. unimolecular decomposition), and 2r2p (i.e. metathesis), accounting for 43%, 35%, and 16% of reactions, respectively (Fig. 2b). The remaining 6% of reactions involve 1r3p and 1r4p (i.e. unimolecular decomposition to three and four products through a single TS), as well as 2r3p and 2r4p reactions. The discovery of TSs connecting large numbers of reactant(s) and products in a single-step again reflects the diversity of reactions in RGD1.

The distribution of the number of heavy atoms in each reaction is another important factor in reaction curation (Fig. 2c). The simplest reactions in RGD1 contain three heavy atoms and the most complex reactions contain ten heavy atoms. Compared with the Grambow dataset in which the system size was limited to seven heavy atoms^2^, 57.8% of reactions in RGD1 contain more than seven heavy atoms. This increase in size is consequential for considering conformational effects on activation energy estimation when using ML models. The number of times each element occurs as a reactive atom (atom A, B, C and D in Fig. 1a) are counted to describe the local graphical features of each reaction (Fig. 2d). Carbon atoms contribute 58% of the reactive atoms while nitrogen atoms contribute 20%. The reactive atom distribution partially reflects the number of bonds each atom can form. For example, hydrogen is typically a terminal atom (one bonded neighbor), so there are a limited number of depth one unique reactions possible compared with carbon. The distribution also reflects the underlying prevalence of distinct bonding configurations in PubChem and the convergence statistics resulting from the TS localization procedure. Similar considerations apply to the distribution of the types of bonds involved in the RGD1 reactions (Fig. 2e), where the four most common bond type changes are C-C, C-N, C-O and C-H. The small amount of O-O reactions is due to relatively small fraction of peroxide compounds in Pubchem.

### Distribution of activation energy and enthalpy of reaction

**Fig. 3:**
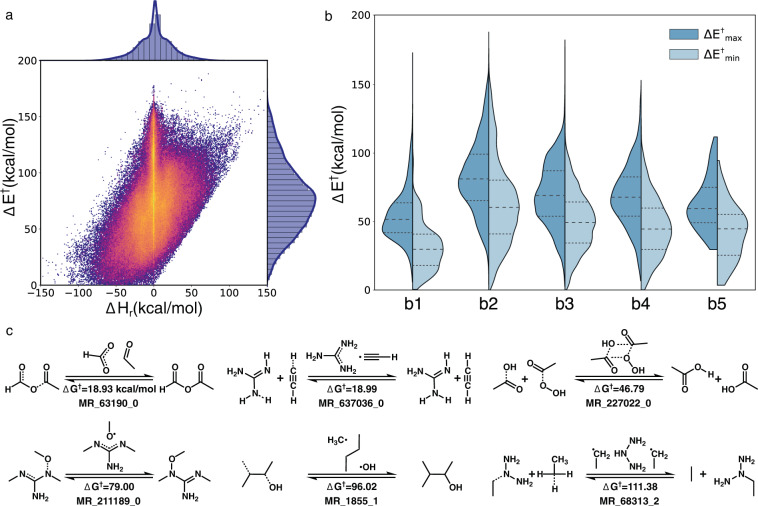
Reaction properties for the RGD1 reactions. (**a**) A bivariate kernel density estimate of the activation energy and enthalpy of reaction for all reactions in the dataset. Both forward and backward reactions are included in this plot. (**b**) The activation energy distribution of reactions corresponding to distinct ERSs. For each reaction, the larger and smaller activation energies of forward and backward direction are considered as ΔE_max_ and ΔE_min_, respectively. (**c**) Six non-trivial reactions in RGD1 with the same reactant and product. The reaction index is provided for each reaction for looking it up in the database.

The property diversity of the RGD1 dataset is illustrated by the overall distribution of activation energies and enthalpies of reaction summarized in the parity plot (Fig. 3). The large range of activation energies (up to 188 kcal/mol) and enthalpies of reaction (from -150 to 150 kcal/mol) span kinetically favored and disfavored as well as endothermic and exothermic reactions. Including positive and negative examples of reactions (many involving the same reactant) is a potential asset of RGD1. Alternatively, users can down-select, say, exothermic reactions in a given application.

The bivariate kernel density estimation reveals a high frequency of Δ*H*_*r*_ = 0 reactions (Fig. 3a,vertical line in the distribution). This subset is composed of 5756 reactions that have identical reactant(s) and product(s). Notably, all these reactions have actual bond changes (as opposed to merely being different conformers) and exhibit a large range of activation energies. Six reactions from this subset that represent different reaction mechanisms are collected in Fig. 3c. In MR_63190_0, the C-O bond breaks first, followed by fragment rotation and formation of a new symmetric C-O bond. A similar reaction mechanism is observed in MR_1855_1 where the exchange of methyl and hydroxyl groups occurs with the rotation of the backbone. For MR_227022_0, the exchange of hydroxyl and peroxide groups occur through a four-membered ring-like TS structure with a relatively low activation energy (46.79 kcal/mol). An even lower activation energy is observed in MR_637036_0, where two hydrogen atoms transfer between a carbon and two nitrogens, resulting in a product that is identical to the reactant due to symmetry. The higher activation energies of MR_211189_0 and MR_68313_2 are associated with the roaming and rotation of larger groups, respectively. These six reactions illustrate the usefulness of this subset of reactions in testing ML models, since many ML featurizations are incapable of predicting the activation energy for reactions with distinct atom-mappings but without net bond-changes.

An extremely weak Bell–Evans–Polanyi (BEP) linear relationship between Δ*H*_*r*_ and Δ*E*^†^ can also be observed in the Fig. 3a parity plot. Given the diversity of the dataset, it is unsurprising that BEP performs poorly, but it is notable since BEP is the most commonly adopted approximation for bypassing direct TS searches. Finally, The linear relationship along the lower edge of the endothermic side of the distribution is trivial, since the activation energy is bound from below by Δ*H*_*r*_.

When decomposing the activation energy distribution based on different ERSs, the average activation energy does not increase monotonically with the number of broken bonds (Fig. 3b). The b1 reactions have the lowest activation energy while the b2 reactions have the highest. This counter-intuitive observation is explained by the reaction database generation process. During reaction enumeration, only b2f2 reactions were considered and reactions corresponding to other ERSs were only discovered through unintended TSs. Since double-ended searches were used to estimate the location of the transition states, unintended reactions needed to be sufficiently favored to intercept the string connecting another reactant-product pair. The relatively low activation energies of b3, b4 and b5 reactions (medium ΔE_max_ less than 75 kcal/mol) is consistent with the increasing kinetic favorability of these unintentionally discovered ERSs.

### Activation energies calculated at different levels of theory

**Fig. 4:**
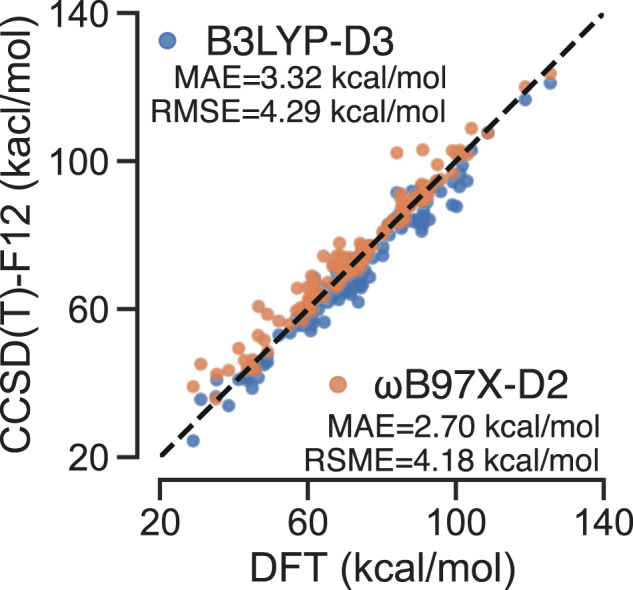
Correlation plot comparing the activation energies calculated at the B3LYP-D3/TZVP (blue) and *ω*B97X-D2/def2-TZVP (orange) with respect to the CCSD(T)-F12/cc-pVDZ-F12//*ω*B97X-D2/def2-TZVP levels of theory.

To assess the significance of the chosen level of DFT theory on the activation energies, a subset of TSs were optimized at both the *ω*B97X-D2/def2-TZVP and B3LYP-D3/TZVP levels used for RGD1, and compared with the single-reference gold-standard method CCSD(T)-F12/cc-pVDZ-F12. For this test set, 127 reactions drawn from three unimolecular decomposition networks were used that have been previously benchmarked at several levels of theory^6^. These 127 reactions contain up to 7 heavy atoms covering the chemical space of C,H,O, and N, which is consistent with RGD1. The previous study benchmarked activation energies calculated at the M05-2X/def2-SVP, *ω*B97X-D2/def2-TZVP and CCSD(T)-F12/cc-pVDZ-F12^41,42^. Geometry optimizations were performed at the first two levels of theory, while single-point calculations were performed at CCSD(T)-F12/cc-pVDZ-F12 level of theory on the geometries optimized by *ω*B97X-D2/def2-TZVP. Here, the geometries were re-optimized and the activation energies were re-evaluated at the B3LYP-D3/TZVP level of theory for comparison with *ω*B97X-D2/def2-TZVP (Fig. 4). The MAE/RMSE of B3LYP-D3 and *ω*B97X-D2 are 3.32/4.29 and 2.70/4.18 kcal/mol, respectively, compared with CCSD(T)-F12. The B3LYP-D3 and *ω*B97X-D2/def2-TZVP functionals systematically underestimate and overestimate the barrier heights by 2.51 and 1.93 kcal/mol, respectively. To help interpret the magnitude of these errors, we note that they are smaller than the errors associated with failing to perform conformational sampling in a recent benchmark that included some of these same reactions^24^. In view of this and the comparable errors of these functionals with respect to the CCSD(T)-F12 results, we consider generating reaction data with a broader representation of elements and reaction mechanisms to be more urgent goals moving forward than reperforming these TS searches with ostensibly more accurate DFT functionals and basis sets.

## Usage Notes

We provide Python scripts for extracting the reaction database and reproducing some of the figures on a public supporting GitHub repository^40^. For example, Code example 1 illustrates how to load the model reaction dataset from a HDF5 file. The “Rind” refers to the reaction index with a format of “MR_XXX_X” and the keys of item Rxn are provided in Table 2.

**Code example 1.** Example of a Python code to load the model reaction database

The uses of the Python scripts are listed below.

- parse_data.py: This script parses all the model reactions stored in a HDF5 file that can be freely downloaded from figshare^38^.
- make_stat_plots.py: This script parses a csv file of reaction features and makes bar plots of different reaction features as shown in Fig. 2.
- draw_violin.py: This script parses csv files of reaction features and DFT energies and makes violin plots of the activation energy distribution of different reaction types as shown in Fig. 3b.

The additional csv files can also be found in the same folder from Github.

## Data Availability

The version of YARP used to generate the data is freely available through GitHub under the GNU GPL-3.0 License^43^. The code used to load the reaction dataset, parse the reaction features and DFT energies, and reproduce Figs. 2, 3 is also freely available^40^. Further details on how to use these scripts are given in the Usage Notes.
